# Correction: Ibarra et al. Selective Photo-Assisted Eradication of Triple-Negative Breast Cancer Cells through Aptamer Decoration of Doped Conjugated Polymer Nanoparticles. *Pharmaceutics* 2022, *14*, 626

**DOI:** 10.3390/pharmaceutics16101281

**Published:** 2024-09-30

**Authors:** Luis Exequiel Ibarra, Simona Camorani, Lisa Agnello, Emilia Pedone, Luciano Pirone, Carlos Alberto Chesta, Rodrigo Emiliano Palacios, Monica Fedele, Laura Cerchia

**Affiliations:** 1 Instituto de Biotecnología Ambiental y Salud (INBIAS), Universidad Nacional de Río Cuarto y CONICET, Río Cuarto X5800BIA, Argentina; 2 Departamento de Biología Molecular, Facultad de Ciencias Exactas, Fisicoquímicas y Naturales, Universidad Nacional de Río Cuarto, Río Cuarto X5800BIA, Argentina; 3 Institute of Experimental Endocrinology and Oncology “G. Salvatore” (IEOS), National Research Council (CNR), 80131 Naples, Italy; s.camorani@ieos.cnr.it (S.C.); lisa.agnello@ieos.cnr.it (L.A.); mfedele@unina.it (M.F.); 4 Institute of Biostructures and Bioimaging, National Research Council (CNR), 80145 Naples, Italy; emiliamaria.pedone@cnr.it (E.P.); luciano.pirone@cnr.it (L.P.); 5 Instituto de Investigaciones en Tecnologías Energéticas y Materiales Avanzados (IITEMA), Universidad Nacional de Rio Cuarto y CONICET, Río Cuarto X5800BIA, Argentina; cchesta@exa.unrc.edu.ar (C.A.C.); rpalacios@exa.unrc.edu.ar (R.E.P.); 6 Departamento de Química, Facultad de Ciencias Exactas, Fisicoquímicas y Naturales, Universidad Nacional de Río Cuarto, Río Cuarto X5800BIA, Argentina

## Error in Figure

In the original publication [1] there was an error in Figure 5b. During the assembly of the image panel, we inadvertently included a duplicate of the control group image representing the CPN-PSMA-sTN29 group (30 min). The correct figure is provided below. The authors state that the scientific conclusions are unaffected. This correction was approved by the Academic Editor. The original publication has also been updated.

## Figures and Tables

**Figure 5:**
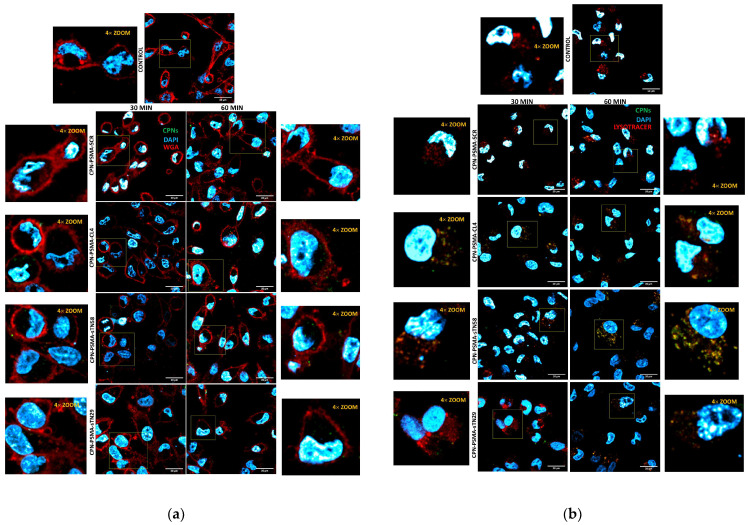
Intracellular localization of aptamer-decorated CPNs in TNBC cells. Representative confocal images of MDA-MB-231/cis cells exposed to 2 mg/L CPNs for 30 and 60 min and stained with WGA for cell membrane visualization (**a**) or LysoTracker Red for lysosome visualization (**b**).

## References

[1] Ibarra L.E., Camorani S., Agnello L., Pedone E., Pirone L., Chesta C.A., Palacios R.E., Fedele M., Cerchia L. (2022). Selective Photo-Assisted Eradication of Triple-Negative Breast Cancer Cells through Aptamer Decoration of Doped Conjugated Polymer Nanoparticles. Pharmaceutics.

