# Supplementary material for: Selective Photo-Assisted Eradication of Triple-Negative Breast Cancer Cells through Aptamer Decoration of Doped Conjugated Polymer Nanoparticles
Source: Pharmaceutics. 2022 Mar 12;14(3):626. doi: 10.3390/pharmaceutics14030626 (PMC8955042; doi:10.3390/pharmaceutics14030626)
Supplement: Supplementary file 1 [file pharmaceutics-14-00626-s001.zip › pharmaceutics-1628067-supplementary.pdf]

pharmaceutics

Supplementary Materials: Selective Photo-Assisted Eradication of Triple-Negative Breast Cancer Cells through Aptamer Decoration of Doped Conjugated Polymer Nanoparticles

Supplementary Results

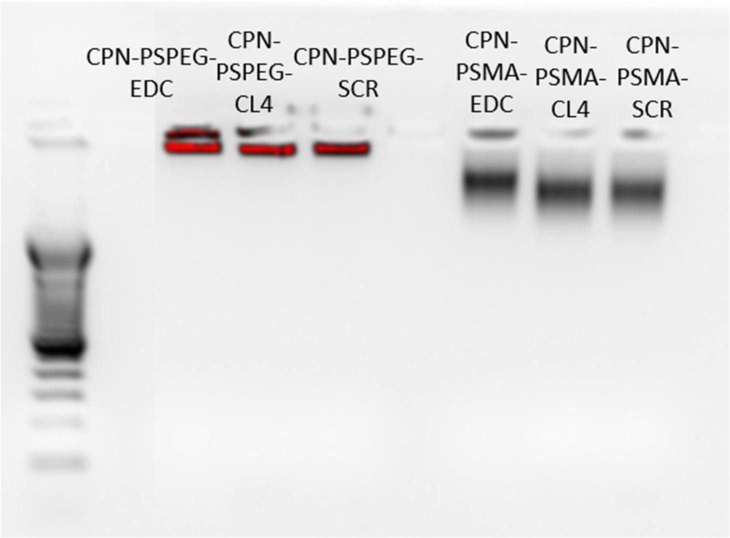

Figure S1. Aptamer-decorated CPN characterization. Gel electrophoresis of different CPN-PSMA and CPN-PSPEG nanoparticles in 1.5 % agarose gel.

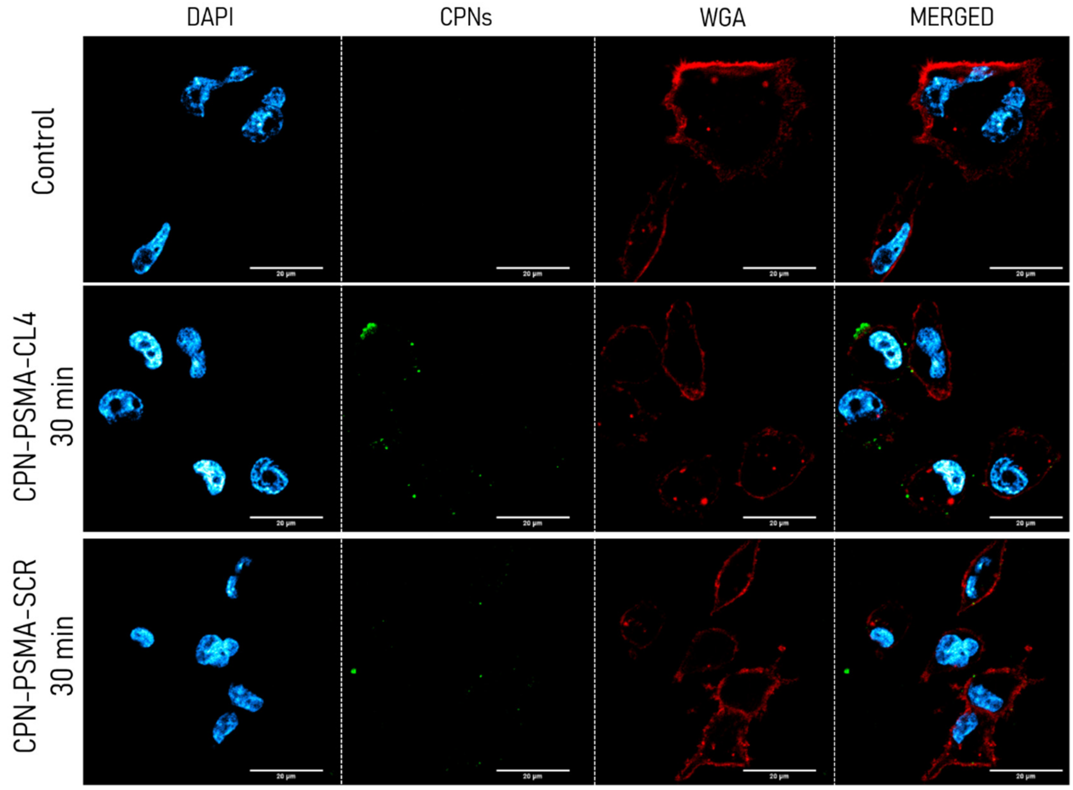

(**a**)

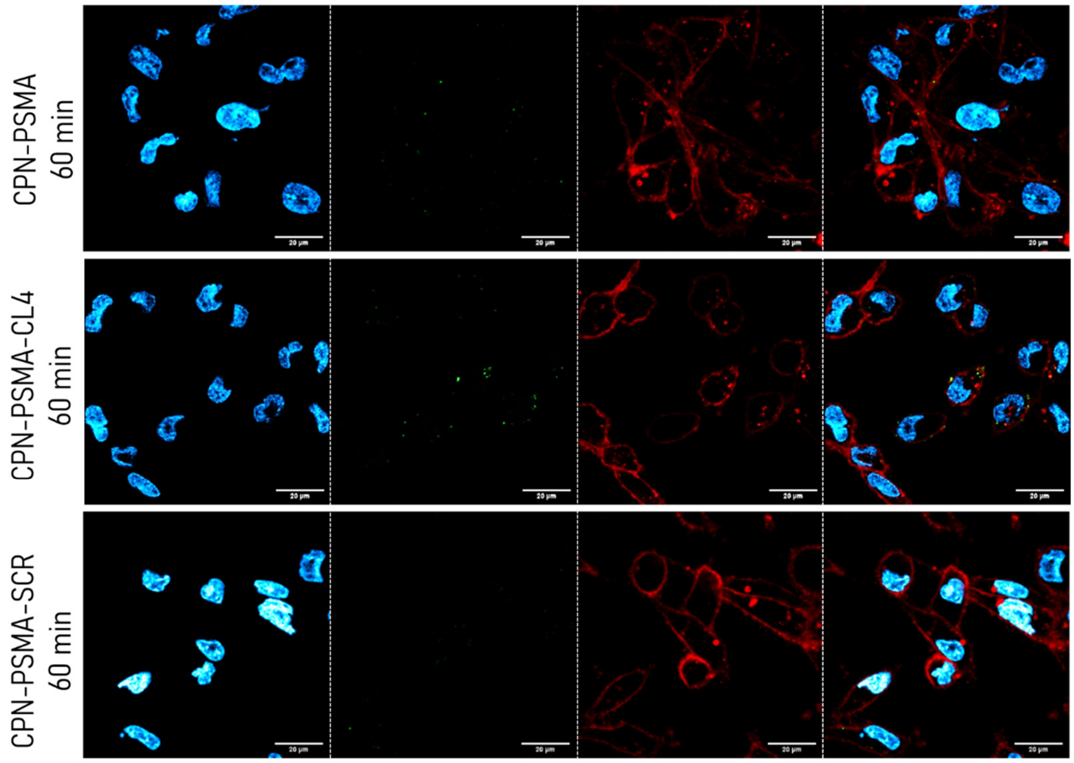

(**b**)

Figure S2. Uptake analysis of aptamer-decorated CPN in TNBC cells. Representative confocal microscopy images of MDA-MB-231 cells exposed to aptamer-decorated CPNs for different periods 30 (a) and 60 min (b) and stained with DAPI (nuclei) and WGA (cell membrane).

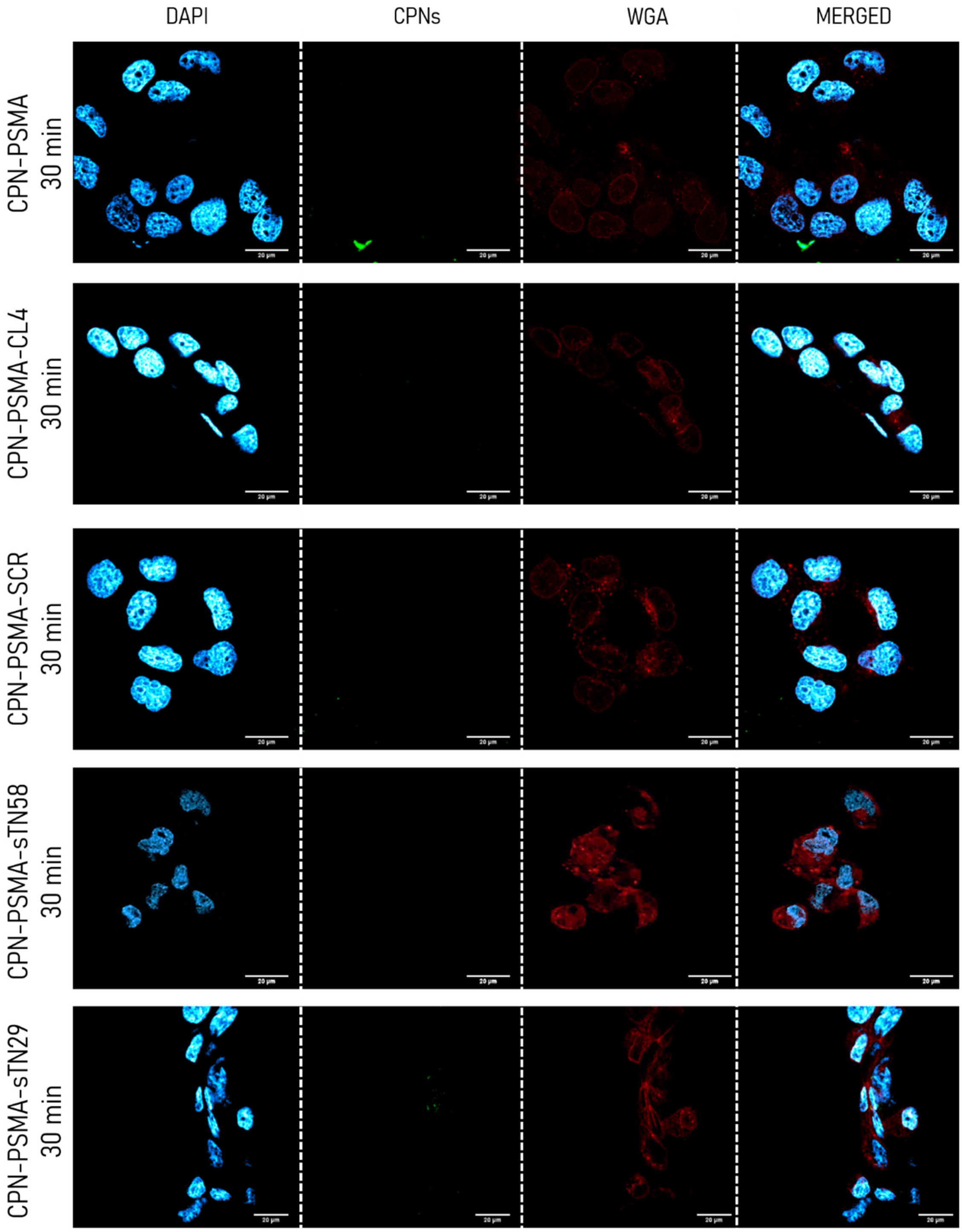

Figure S3. Uptake analysis of aptamer-decorated CPN in TPBC cells. Representative confocal microscopy images of non-TNBC BT-474 cells exposed to aptamer-decorated CPNs for 30 min and stained with DAPI (nuclei) and WGA (cell membrane).

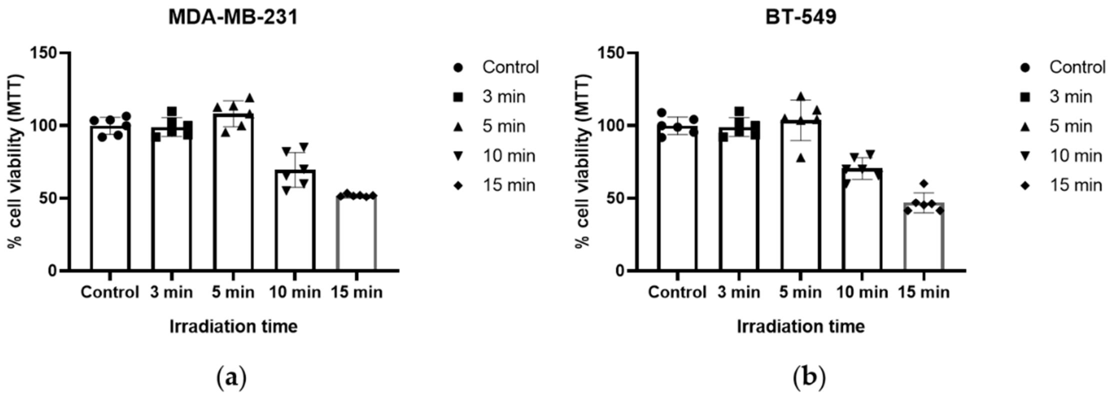

Figure S4. Light irradiance evaluation in TNBC cells. Cell viability in MDA-MB-231 (a) and BT-549 (b) cells exposed to light irradiance for different times at 50 mW/cm² of potency.

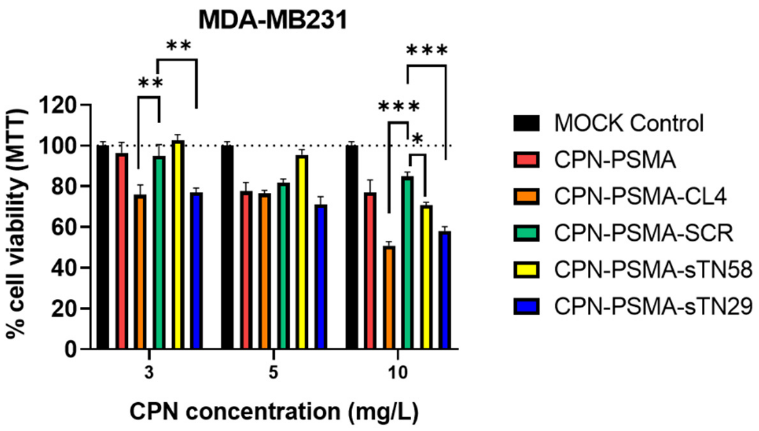

Figure S5. PDT efficacy with aptamer-decorated CPNs in TNBC cells. Cell viability in TNBC MDA-MB231 after 24 h of PDT with increasing concentration of different aptamer-decorated CPN-PSMA and unconjugated CPN-PSMA and a light dose of 10 J/cm².
